# Post-drought hydraulic recovery is accompanied by non-structural carbohydrate depletion in the stem wood of Norway spruce saplings

**DOI:** 10.1038/s41598-017-14645-w

**Published:** 2017-10-30

**Authors:** Martina Tomasella, Karl-Heinz Häberle, Andrea Nardini, Benjamin Hesse, Anna Machlet, Rainer Matyssek

**Affiliations:** 1 Department of Ecology and Ecosystem Management- Chair for Ecophysiology of Plants, Technische Universität München, Hans-Carl-von-Carlowitz Platz 2, 85354 Freising, Germany; 2 Department of Life Sciences, Università degli Studi di Trieste, Via L. Giorgieri 10, 34127 Trieste, Italy

## Abstract

Hydraulic failure and carbon starvation are recognized as main causes of drought-induced forest decline. As water transport and carbon dynamics are strictly interdependent, it is necessary to clarify how dehydration-rehydration cycles are affecting the relations between stem embolism and non-structural carbohydrates (NSC). This is particularly needed for conifers whose embolism repair capability is still controversial. Potted Norway spruce saplings underwent two drought-re-irrigation cycles of same intensity, but performed in two consecutive summers. During the second cycle, stem percent loss of hydraulic conductivity (PLC) and NSC content showed no carry-over effects from the previous drought, indicating complete long-term recovery. The second drought treatment induced moderate PLC (20%) and did not affect total NSCs content, while starch was converted to soluble sugars in the bark. After one week of re-irrigation, PLC recovered to pre-stress values (0%) and NSCs were depleted, only in the wood, by about 30%. Our data suggest that spruce can repair xylem embolism and that, when water is newly available, NSCs stored in xylem parenchyma can be mobilized over short term to sustain respiration and/or for processes involved in xylem transport restoration. This, however, might imply dependency on sapwood NSC reserves for survival, especially if frequent drought spells occur.

## Introduction

Increase in duration, intensity and frequency of dry spells, coupled with rising temperatures, enhance drought stress experienced by plants^1^. Water shortage in trees causes impairment of long distance water transport when critically high xylem tensions induce embolism formation and spread^2^, but can also affect carbon relations and metabolism. In fact, stomatal regulation of leaf transpiration during drought can significantly decrease carbon assimilation rates and, in extreme cases (e.g. mild but prolonged droughts), lead to negative net carbon balance^3^, when trees must necessarily rely on non-structural carbohydrate (NSC) reserves^4^ to maintain metabolic processes. On the other hand, dehydration can reduce mobilization of carbon^5^, modify carbon allocation^6^, affect phloem functioning^7,8^ and compromise enzymatic activities involved in sugars metabolism (e.g. starch hydrolysis^9^). Due to the complexity of interactions between variables affecting NSC pools during drought (in particular duration and intensity of drought episodes^10^), and to species-specific water-use strategies^11^, a strong evidence of whether, how and in which compartments NSC reserves are affected in tree species is needed^12,13^.

There is proof that stored NSCs and their metabolism are also involved in maintaining and restoring xylem transport capacity, allowing for water refilling of gas-filled conduits^14–16^. In the current model proposed for embolism repair, developed during the past few decades^17^, soluble sugars are transferred from parenchyma cells (i.e. vessel associated cells, VACs) into embolized xylem ducts, in order to establish an osmotic gradient that reclaims water from VACs and/or phloem^18^ to the conduits and allows for repair. This mechanism requires the presence of living cells in the proximity of the embolized conduits, so that both spatial arrangement and amount of woody parenchyma would therefore be important traits affecting the process^19^. For this reason woody angiosperms, having an average parenchyma fraction in the secondary xylem of about 26% against an average of 8% calculated in conifers^20^, are more likely to show active post-drought hydraulic recovery than conifers^17,21,22^. On the other hand, pit membranes of conifers have a torus-margo structure: when embolism occurs, the torus is aspirated to the pit chamber aperture, operating as a sealing valve and isolating the embolized tracheids from adjacent functional ones^23^. As isolation of gas-filled conduits is required during refilling under negative pressures^24^, in conifers the repair of embolized conduits would be favoured by this sealing mechanism^25^.

In conifers, studies on repair of drought-induced embolism are generally lacking. Refilling has been demonstrated to occur in some conifers after frost-induced embolism via needle water uptake upon thawing of snow^25^ or after drought-induced embolism via needle cuticle^26^ or bark^27,28^ water absorption. Evidence supporting the involvement of active refilling in conifers is scant^29–32^, while some studies have shown no repair^22,33^ or have reported cambial re-growth as the main strategy for partial recovery of water transport^34^.

In this study we present an experiment performed on Norway spruce (*Picea abies*), a conifer currently threatened by climate change in Central Europe^35^. Spruce is known to be relatively isohydric, i.e. leaves have an efficient stomatal regulation of water loss under drought that allows for the maintenance of water potentials above cavitation thresholds for long periods^36^. On the other hand, this strategy would theoretically put this species at risk of carbon stores depletion, especially under prolonged and mild droughts^3,10^. In previous studies conducted on this species, drought has been reported to affect carbon allocation and translocation, with differences between aboveground and belowground organs^5,6^.

**Figure 1:**
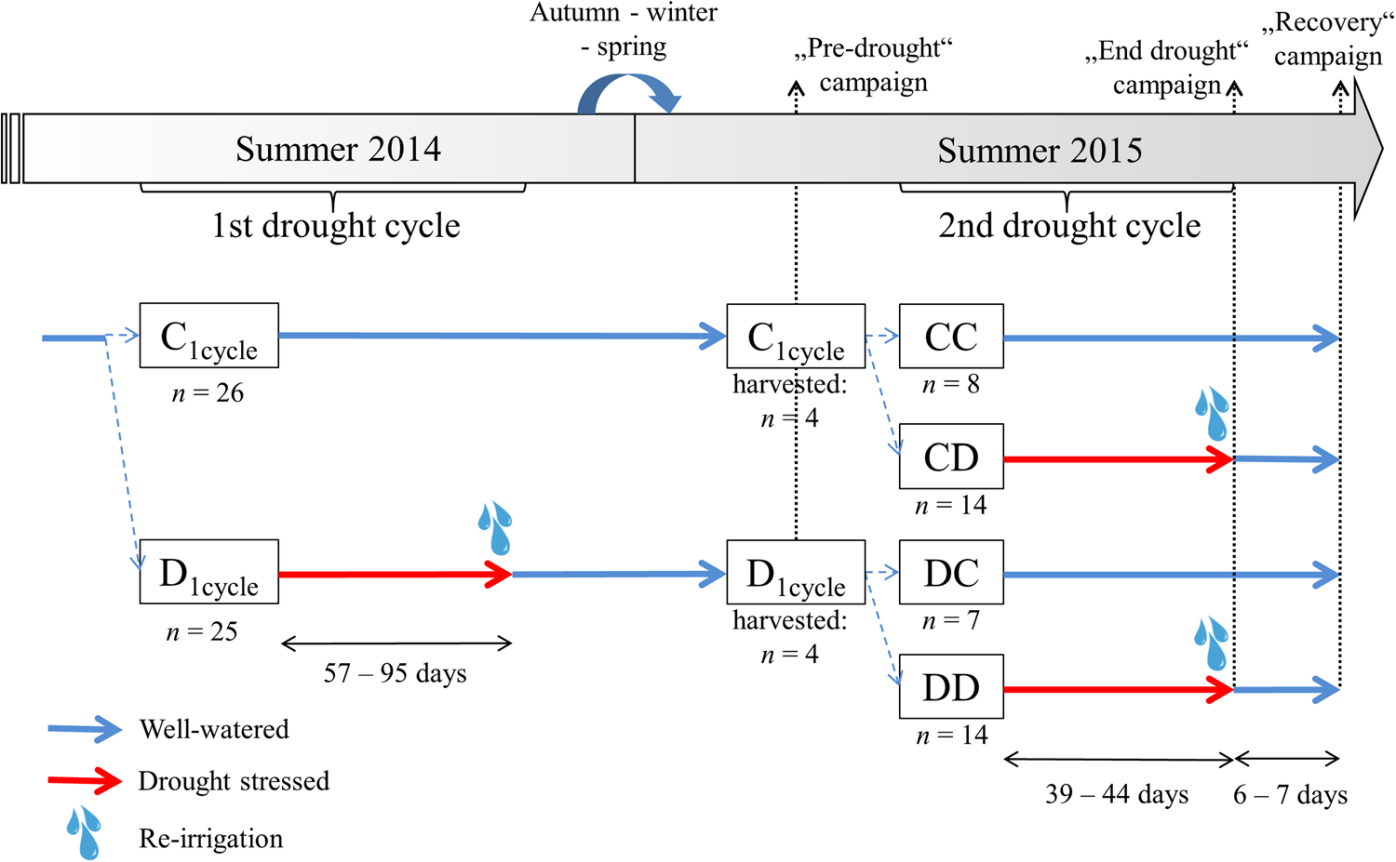
Scheme of the experimental setup. In the first cycle (summer 2014), spruce potted trees were split into two treatments: a control (C_1cycle_), kept well watered, and drought stressed one (D_1cycle_). In both drought cycles, midday leaf water potentials (Ψ_md_) were periodically monitored and plants were dehydrated to a target Ψ_md_ (between −3 and −3.5 MPa), at which each single plant was re-irrigated. During the first drought cycle, irrigation was completely withheld in D_1cycle_ plants. After re-irrigation, all plants were kept well-watered until the following summer (2015), when the second drought cycle took place. In summer 2015 a first measurement campaign (“Pre-drought”) was performed: C_1cycle_ and D_1cycle_ plants, all well-watered, were compared to check for carry-over effects of the first drought cycle. Afterwards, both C_1cycle_ and D_1cycle_ groups were divided into two, a well-irrigated and a drought-stressed one. Therefore, four groups were formed (CC, CD, DC and DD). In this second drought, CD and DD plants were dehydrated progressively, regulating the amount of irrigation in order to reach the target Ψ_md_ almost at the same time (within a few days). At the peak of drought and after one week of re-irrigation, a second (“End drought”) and a third (“Recovery’’) measuring campaigns were performed, respectively. In the three measuring campaigns performed in the second drought cycle, predawn (Ψ_pd_), xylem (Ψ_xyl_) and midday (Ψ_md_) water potentials, percent loss of stem hydraulic conductance (PLC) and stem non-structural carbohydrates (NSC) were measured.

Water relation parameters and embolism dynamics during drought-irrigation cycles have been studied in several angiosperm species^37–40^. Nevertheless, only a few studies related drought and subsequent recovery of stem hydraulics with stem NSCs content^39,40^. In some angiosperm species, a strong association between embolism dynamics (formation and removal) and conversion of NSCs^39^ has been reported and stem xylem embolism reversal correlated with the amount of soluble carbohydrates at the end of the drought period^40^. In conifers, this type of approach is generally missing. In our study, potted juvenile spruce trees were subjected to two drought-re-irrigation cycles performed in two consecutive growing seasons (summer 2014 and 2015). While in the first cycle only two treatments were present (control, C_1cycle_, kept well watered, and drought stressed, D_1cycle_), for the second cycle each group was split into two (control and drought), so that four groups were formed and compared (CC, CD, DC and DD; see Fig. 1). Both drought cycles aimed at reaching midday leaf water potentials (Ψ_md_) between −3 and −3.5 MPa, after which plants were re-irrigated. These thresholds correspond for Norway spruce to embolism levels below theoretical lethal thresholds for conifers (i.e. below 50% percent loss of xylem hydraulic conductance, PLC^34,41^). In the second drought cycle (summer 2015), coupling water relations and hydraulic measurements with stem NSCs content, detected separately in wood and bark, we analyzed: (1) possible long-term (or carry-over) effects of the first year drought; (2) stem NSCs dynamics under drought, as possibly influenced by the species’ isohydric behaviour; (3) the capability to recover xylem hydraulics in the short period (days) after re-irrigation; (4) possible relations between hydraulic recovery and stem NSC content variations. We tested the hypotheses that in spruce: (1) in the long term, the previous drought event affects NSC pool size; (2) at the end of drought stem NSCs are depleted, due to the relatively isohydric strategy of the species; upon re-irrigation (3) hydraulic function is rapidly recovered and (4) stem NSCs content decreases because, in line with the current model for refilling^17^, sugars previously accumulated in the xylem apoplast would be washed away when the xylem conduits are refilled and water transport is restored.

## Results

### Environmental and water relations data

In the first drought cycle (summer 2014), plants reached the target midday water potentials (Ψ_md_, average −3.24 ± 0.04 MPa) in 57 to 95 days (see Supplementary Fig. S1). In the following summer, under well-watered conditions (i.e. in the “Pre-drought” campaign, end June 2015), no significant effects were found in leaf water relations, except for xylem water potentials (Ψ_xyl_), which were less negative in previously stressed plants (i.e. in D_1cycle_, *P* = 0.01, Supplementary Table S1).

**Figure 2:**
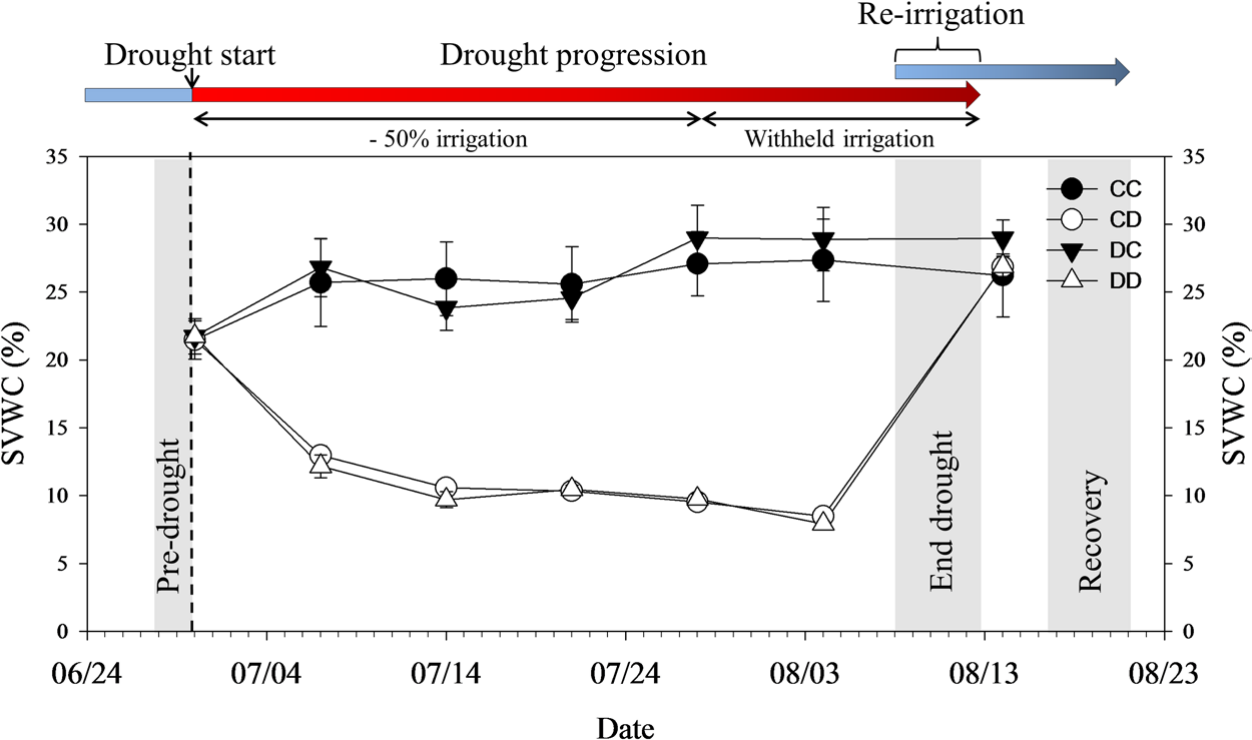
Soil volumetric water content (SVWC) measured over time during the second drought cycle (summer 2015). CC = control in 2014 and 2015; CD = control in 2014, drought in 2015; DC = drought in 2014, control in 2015; DD = drought in 2014 and 2015. Symbols are means and error bars denote standard errors (*n* = 3–14 per treatment and campaign). The dashed vertical line shows the beginning of the second drought cycle and the three shaded areas highlight the time periods when, in order of time, the “Pre-drought”, “End drought” and “Recovery” campaigns were performed. Above the graph, the irrigation regime for drought stressed plants is explained: in the first four weeks of drought, drought plants were irrigated with half of their individual daily water consumption; afterwards irrigation was completely withheld until re-irrigation.

**Figure 3:**
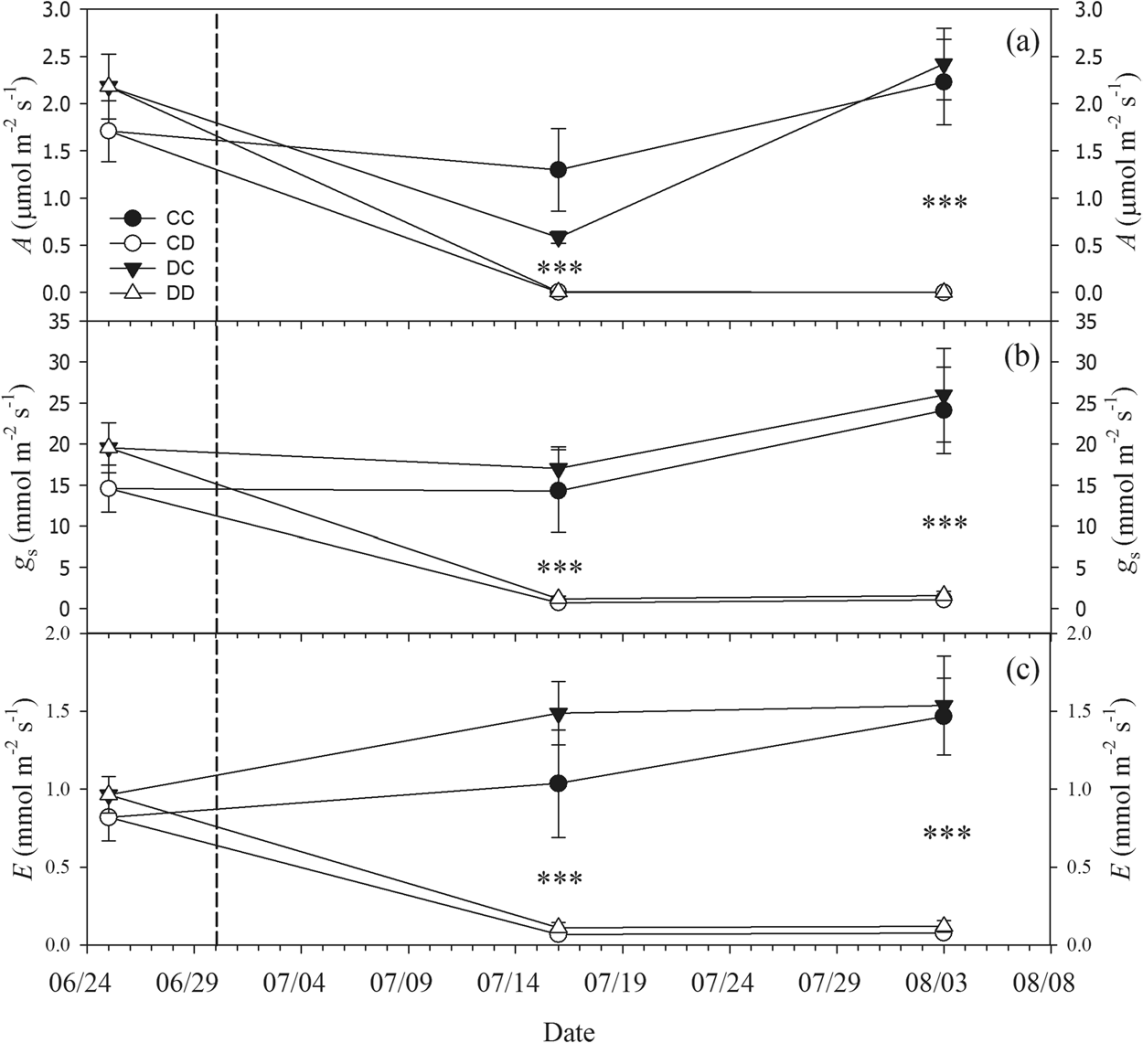
Gas exchange rates measured over time, before (25 June, “Pre-drought” campaign) and throughout the second drought cycle (summer 2015). (**a**) CO_2_ assimilation rate (*A*), (**b**) stomatal conductance (*g*_s_) and (**c**) leaf transpiration (*E*). The beginning of the drought treatment is represented by the vertical dashed line. CC = control in 2014 and 2015; CD = control in 2014, drought in 2015; DC = drought in 2014, control in 2015; DD = drought in 2014 and 2015. Symbols are means, error bars denote standard errors and asterisks indicate significant differences (*P* < 0.001) between drought (CD and DD) and control (CC and DC, well-watered throughout the whole drought cycle) treatments (Kruskal-Wallis test and Conover’s post-hoc for *A*, one-way ANOVA and Tukey-HSD for *g*_s_ and *E*).

During the second drought cycle, the soil water content (SVWC) in the two drought treatments (CD and DD) gradually decreased, reaching 8% in the last week of the experiment (Fig. 2). Gas exchange parameters (*A*, *g*_s_ and *E*) measured in drought stressed plants were close to zero after only two weeks of drought, while control plants maintained over the whole period almost constant values. No difference in gas exchange was found between the two control (CC and DC) as well as between the two drought (CD and DD) treatments (Fig. 3).

**Figure 4:**
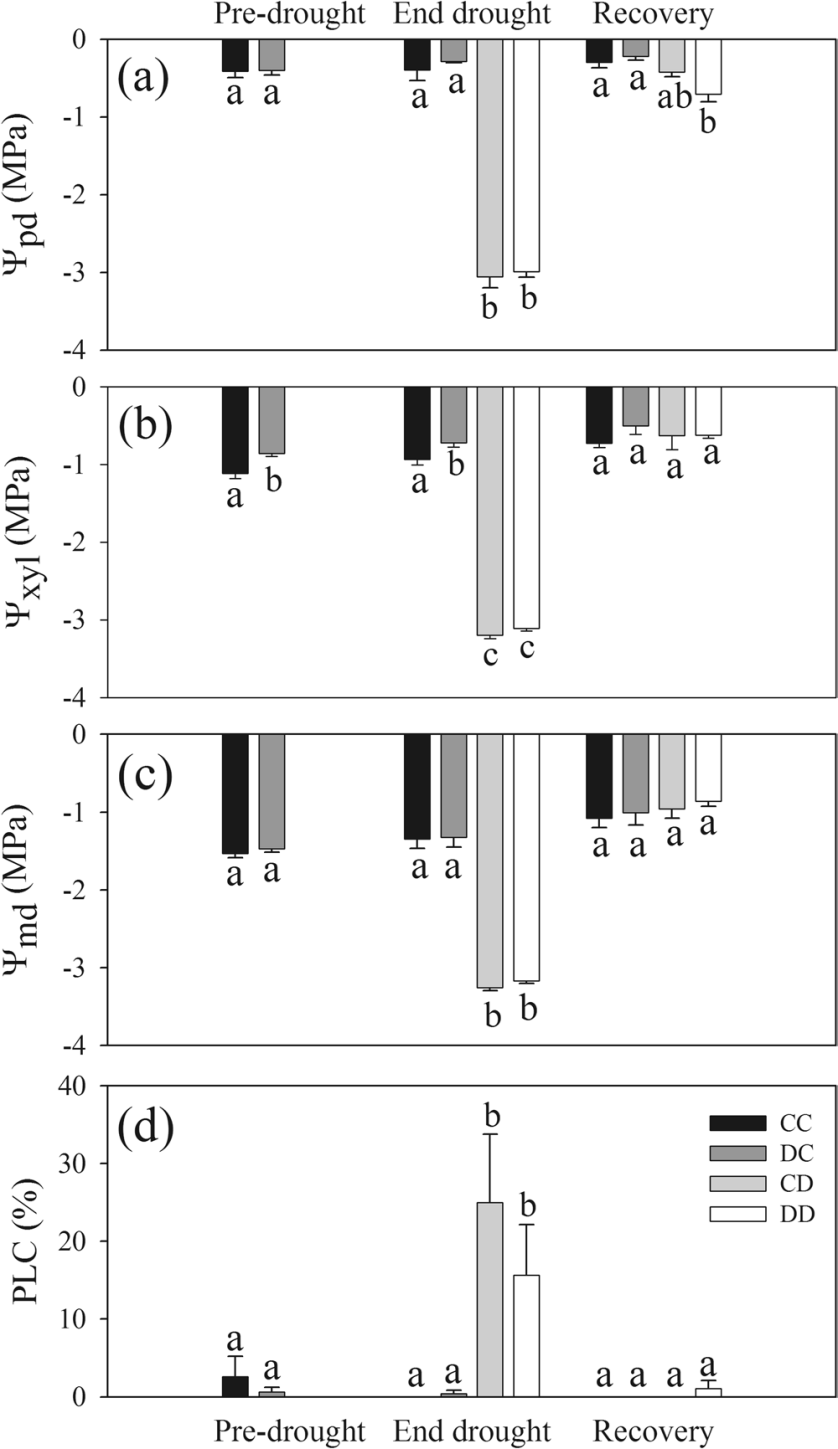
Water potentials and xylem embolism dynamics in the second drought cycle (2015). (**a**) Predawn (Ψ_pd_), (**b**) xylem (Ψ_xyl_) and (**c**) midday water potentials (Ψ_md_) and (**d**) percentage loss of stem xylem hydraulic conductance (PLC) measured in “Pre-drought”, “End drought” and “Recovery” campaigns. CC = control in 2014 and 2015; CD = control in 2014, drought in 2015; DC = drought in 2014, control in 2015; DD = drought in 2014 and 2015. Bars are means ± standard error. Different letters indicate differences among treatments within each campaign. The “Pre-drought” campaign shows values measured in well-watered plants before that C_1cycle_ and D_1cycle_ would have been split into two groups (for explanation see Fig. 1 and methods part).

The two drought treatments reached the target Ψ_md_ (average of about −3.2 MPa; range from −3.0 MPa to −3.5 MPa) after 39 to 44 days of drought and pre-dawn water potentials (Ψ_pd_) and Ψ_xyl_ were close to Ψ_md_ in both groups (Fig. 4a and b). At the end of the drought cycle, both control treatments maintained Ψ_md_ at about −1.3 MPa and Ψ_xyl_ was less negative in DC than in CC plants (*P* = 0.03, Fig. 4b). Upon re-irrigation, water potential isotherm parameters and LMA (Table S2, supplementary material) did not differ between irrigated and stressed plants. On 16 August, after re-irrigation, Ψ_pd_ were about 0.5 MPa lower in DD plants than in the two control groups (about −0.2 MPa) and CD plants showed intermediate values (Fig. 4a). Six to seven days after re-irrigation, Ψ_xyl_ and Ψ_md_ were similar to control values (about −0.6 MPa and −1.0 MPa, respectively; Fig. 4b,c).

### Native xylem embolism

In the second drought cycle (summer 2015), percentage loss of stem xylem hydraulic conductance (PLC) measured in well-watered plants, was close to zero (Fig. 4d). Drought induced a significant increase in PLC, and at the end of the drought period (“End drought” campaign) mean values of about 25% and 15% were measured in CD and DD trees, respectively, with no significant difference between the two treatments (*P* = 0.81). After irrigation, drought stressed plants showed complete recovery of PLC to pre-stress values (i.e. close to zero, Fig. 4d).

### Stem diameter variation

**Figure 5:**
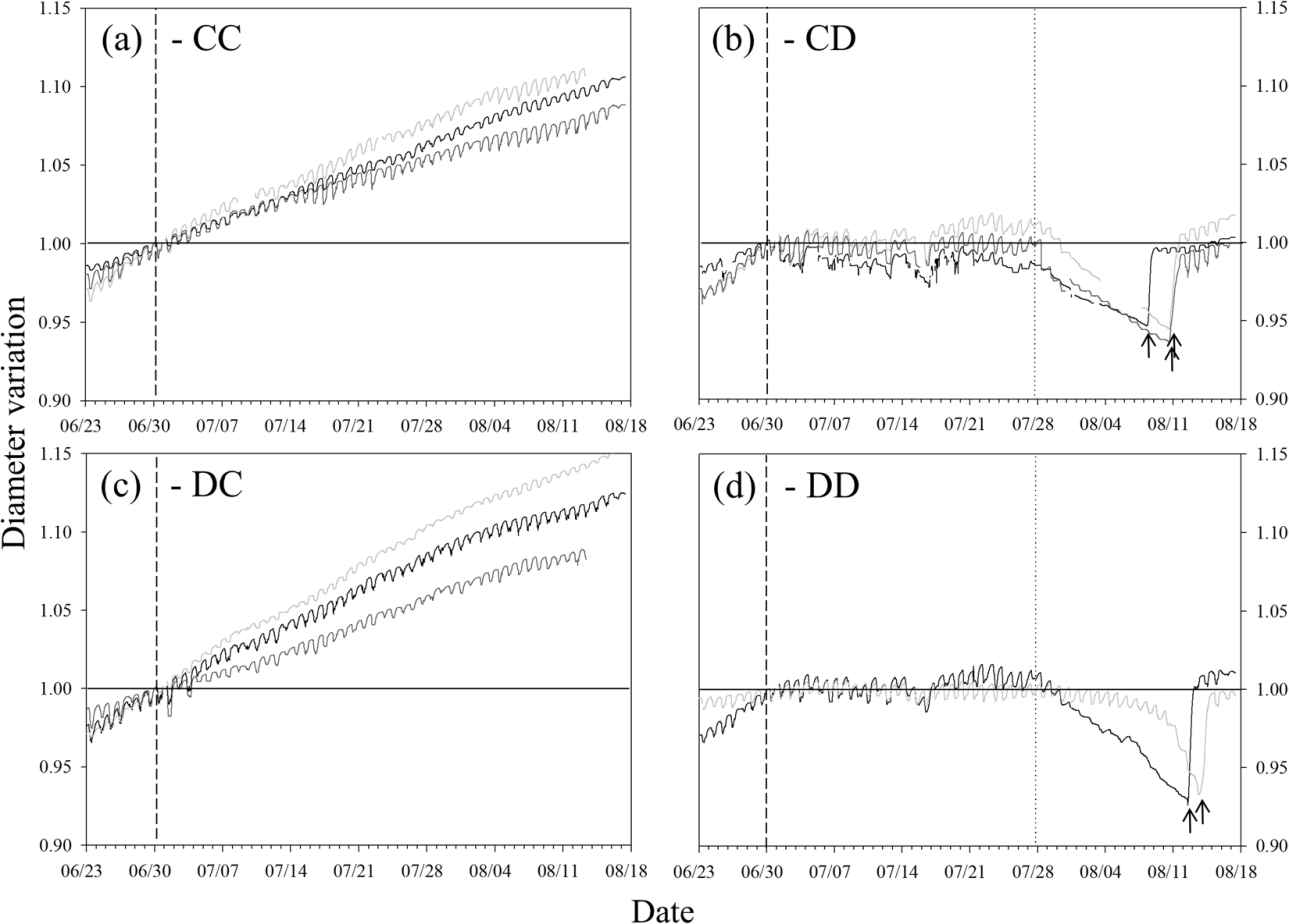
Stem diameter variation over the second drought cycle (summer 2015). Stem diameter variations measured in **(a**) CC (control in 2014 and 2015), (**b)** CD (control in 2014, drought in 2015), **(c)** DC (drought in 2014, control in 201) and **(d)** DD (drought in 2014 and 2015) treatments. Values are expressed as stem diameter variation from the diameter measured at the beginning of the drought treatment at the basal stem of two-three trees per treatment. Different line colours indicate different tree individuals. Vertical dashed and dotted lines show, respectively, the beginning of the drought treatment and the time when irrigation was completely withheld in drought trees. Arrows indicate the time when each tree individual was re-irrigated after drought.

During the second drought cycle (summer 2015), control trees showed a continuous increase in stem diameter (Fig. 5a,c). In both drought treatments (CD and DD), water shortage induced the complete stop (or a slight reduction) of radial growth. While in the first 30 days of drought diurnal cycles of shrinking-swelling were still maintained (Fig. 5b,d), in the final phase of drought (about 10 days before re-irrigation), when irrigation was completely stopped, daily fluctuations were almost negligible and a pronounced shrinkage was observed. Upon re-irrigation, stems of drought trees started to gradually swell and diameters reached the pre-stress values after one day.

### Stem non-structural carbohydrate content

**Figure 6:**
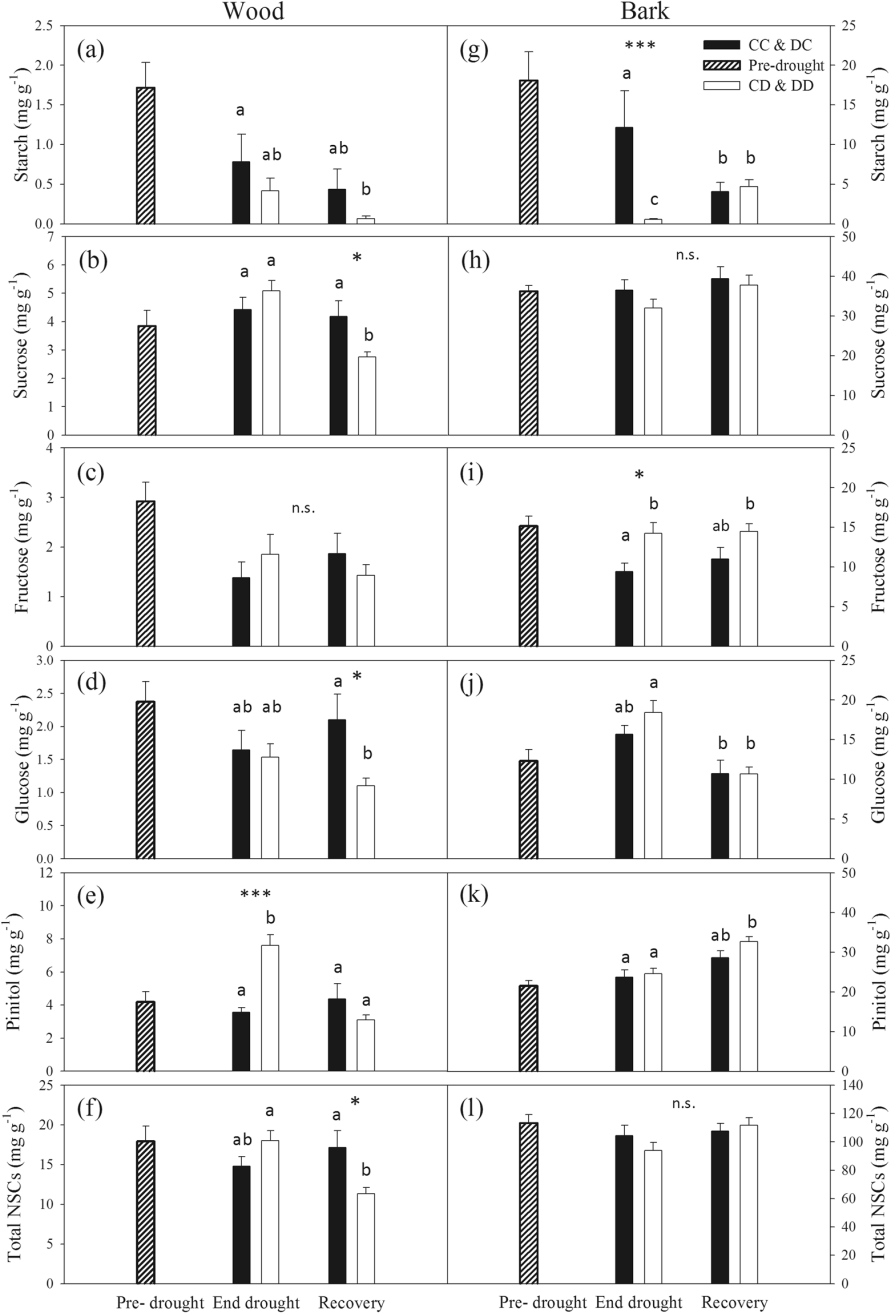
Non-structural carbohydrate (NSC) dynamics in stems during the second drought cycle (2015). (**a**–**f**) NSC concentration (in mg g^−1^ of dry mass) in stem wood and **(g**–**l)** bark, measured in “Pre-drought”, “End drought” and “Recovery” campaigns. In the “Pre-drought” campaign, data were pooled in one single group (*n* = 8). In the “End drought” and “Recovery” campaigns, well-watered (CC and DC) as well as drought (CD and DD) treatments were pooled, resulting in one control (CC & DC) and one drought (CD & DD) group. Please note the different scales between wood and bark NSCs content. “Total NSCs” is the sum of all NSC specimens (starch, sucrose, fructose, glucose, pinitol, stachyose, raffinose and galactose) analysed. Bars are means ± standard error (*n* = 6–12). Different letters indicate significant differences between treatments and campaigns (two-way ANOVA and Tukey-HSD, only data of “End drought” and “Recovery” are compared). n.s. = no significant difference. Asterisks denote the significance of differences among treatments within a given campaign (*0.01 < *P* < 0.05, **0.001 < *P* < 0.01, ***P < 0.001).

The first drought cycle did not influence neither the NSC content measured under well-watered conditions in the “Pre-drought” campaign of 2015 (Supplementary Table S1), nor most of NSC specimens concentration measured in the “End-drought” and “Recovery” campaigns of 2015 (Supplementary Table S3). Only sucrose concentration measured in the bark at the end of drought was higher (*P* = 0.03) in plants stressed the year before. Most differences in NSC content measured at the end of drought and upon re-irrigation could be ascribed to the current year drought (i.e. second drought cycle, Table S3). Hence, for sake of clarity, NSC data of the two control treatments (CC and DC) as well as those of the two drought treatments (CD and DD) were pooled together (Fig. 6 and Supplementary S2).

NSC content was much (about ten-folds) higher in the stem bark than in the wood. In the wood, drought significantly affected only pinitol content (*P* < 0.001), which doubled to ca. 8 mg g^−1^, and stachyose content (*P* = 0.03, Supplementary Fig. S2). In the bark, at the end of drought, starch was almost completely depleted (average of 0.6 mg g^−1^) in drought stressed trees and fructose content was about 50% higher than in control trees (Fig. 6). In both wood and bark, the drought treatment did not affect the amount of total soluble sugars as well as the total NSC content.

After one week of re-irrigation (“Recovery” campaign), the content of NSC specimens measured in the wood of drought stressed trees decreased: sucrose, glucose and total NSC content were depleted by about 30% (Fig. 6). In the bark, no difference between treatments was detected at recovery, for all main NSC specimens (Fig. 6).

PLC measured at the end of drought in drought stressed trees negatively correlated with the respective starch concentration in the wood (ρ  = −0.528, *P* = 0.024), but did not correlate with soluble sugars content (ρ  = 0.222, *P* = 0.375; see also Fig. S3, Supplementary material).

### Aboveground biomass

Only the first year drought cycle substantially (*P* = 0.060) affected trees aboveground biomass, which at the end of the experiment (August 2015) was 137 ± 14 g in C_1cycle_ and 103 ± 9 g in D_1cycle_ trees.

## Discussion

Long-lasting droughts are supposed to induce carbon depletion, especially in relatively isohydric species like Norway spruce^3^. The first drought cycle, which lasted two to three months, did not induce in the studied spruce saplings any relevant long term impairment of water transport and, most importantly, stem NSC pool size. The new functional xylem built in spring from the cambium, is known to contribute to restore xylem functionality in case that residual embolism is still present after winter^42^. Nevertheless, it is likely that, similarly to what we observed in the second drought cycle (see discussion below), a fast hydraulic restoration could have occurred already upon re-irrigation, after the first drought treatment in 2014. Even during the second drought cycle, no carry-over effect was observed, indicating for spruce complete resilience to the drought stress undergone the previous year.

Under drought stress, spruce showed a typical isohydric behaviour: the almost complete stomatal closure observed in the second drought cycle allowed for reduction of leaf water loss, albeit at the expense of carbon assimilation (Fig. 3). It is very likely that high temperatures registered in summer 2015, induced fast dehydration (e.g. in comparison with the 2014 drought), which was additionally exacerbated in the last two weeks of drought when irrigation was completely withheld. The target water potential range reached at the end of the drought treatment induced xylem embolism in the main stem: the measured percentage loss of xylem conductance (PLC) was in the range expected from xylem vulnerability curves reported for the species in the literature^43,44^.

Contrary to our hypothesis, the drought treatment applied in 2015 did not alter stem carbon balance, in both wood and bark compartments (Fig. 6 and Supplementary S2). There is evidence that short-term and severe droughts, like the one undergone by the spruce saplings in 2015, can lead to little or no changes in NSC pool sizes even in relatively isohydric species^45^. It must be noted that stem diameter growth stopped at the time when drought started (Fig. 5b,d), thus likely reducing carbon sink consumption and helping to preserve carbon pools when CO_2_ uptake was drastically reduced. It is also possible that NSC depletion occurred in other tree compartments (e.g. in roots^5,6,46^). Nevertheless, while total NSC content did not change, starch in the bark was almost completely depleted by drought and was accompanied by an increase in monosaccharides (glucose and in particular fructose). This could be an indication of osmotic adjustment of turgor loss point^5,47^. Moreover, there are suggestions that solutes accumulated in the phloem might also be effective for refilling if they are delivered to the VACs through parenchyma rays^16–18^. The consumption of local starch reserves under drought could be also a consequence of impeded phloem translocation^5,12^ and/or reduced sugar availability at the source (i.e. in the leaves) due to photosynthesis inhibition (Fig. 2a).

Some NSC compounds are known to be involved in plant cell protection (as antioxidant and/or osmoprotectant) under stresses like drought. The relevant accumulation of pinitol in the wood of stressed plants at the end of drought, accompanied by a subsequent decrease to pre-stress values upon re-irrigation (Supplementary Fig. S2), can be explained by the role played by this sugar alcohol as hydroxyl radical scavenger and osmolite in some plant species, including conifers^48^.

In our experiment, spruce plants experienced water potentials which were low enough to induce xylem embolism, but still far from those theoretically endangering conifers’ survival (PLC below the threshold of 50%, Fig. 4). In Norway spruce, multiple drying-rewetting cycles, where plants were dehydrated to minimum leaf water potentials slightly below our target values, caused complete and short-term recovery of sapflow rates and stem diameter (as here observed, Fig. 5) in each re-irrigation episode^5^. In our study we obtained evidence that this species is able to recover xylem hydraulics in the main stem over short term after re-irrigation (six-seven days, Fig. 4c). We can exclude the possibility that water uptake via needle cuticle or bark, previously reported in some conifer species^26–28^, could have been responsible for refilling of spruce stems. In fact, relative humidity during the recovery time of the experiment never exceeded 81%, while aboveground uptake is supposed to occur during fog (RH of 100%) or rain events. Also the possibility that newly built xylem conduits (new growth upon re-irrigation) would have allowed for fast hydraulic recovery, can be excluded by dendrometer data (no regrowth, Fig. 5) and cannot explain the observed complete hydraulic recovery. Studies involving *in vivo* imaging have reported the occurrence of refilling under low xylem pressures^49^ and proved in *Vitis riparia* that positive root pressure was not required as a driving force of the process^50^. Positive root pressure, which requires maximum soil water availability (i.e. Ψ_pd_ ~ 0) and complete stop of transpiraton, has been shown to contribute to refilling in some monocots (e.g. in bamboo^51^) or in some woody angiosperms prior spring flush^19,52^, whereas in conifers this phenomenon has not been detected so far. Pre-dawn water potentials at recovery show that embolism repair occurred at relatively high xylem tensions (Fig. 4a) and therefore do not support the involvement of root pressure in embolism removal in our trees.

One of our main working hypotheses was that recovery of xylem hydraulics should be accompanied by changes in NSC content in stem wood and/or bark. Upon hydraulic recovery, NSCs of drought plants were depleted only in the wood, but remained unaltered in the bark (Fig. 6). This suggests that the NSC pool in wood parenchyma was partially degraded upon drought relief, and was still not recharged one week later. We propose some scenarios which might explain our findings. After re-irrigation, NSCs could have been used in the cambium as source of carbon to sustain new cell formation (albeit re-growth was not detected by dendrometers, Fig. 3). This hypothesis, however, would not provide an explanation for sugar depletion only in the wood and not in the bark. Alternatively, sugar reserves might be used to supply carbon demand for stem respiration after re-irrigation, especially if photosynthesis is still depressed. Although we did not measure gas exchange rates after re-irrigation, a delay in recovery of photosynthesis after drought has been previously reported^34,41^. Moreover, an increase in stem respiration at re-irrigation has been associated with an increase in energy demand for xylem transport restoration^39^.

According to the current paradigm for embolism refilling, soluble sugars released by the wood parenchyma into embolized conduits would drive water entry after drought relief^14–16^. If this happened in spruce, soluble sugars could have first entered the embolized tracheids and then, once the conduits had been refilled and become newly functional, removed by the transpiration stream^53^. This mechanism could also explain the drop in starch content in drought stressed trees, as its breakdown could have contributed to generate the osmotic gradient for refilling. From our data we cannot unequivocally relate NSC depletion to active refilling of embolized conduits. Nevertheless, considering the Van’t Hoff’s equation, it is possible to estimate the theoretical minimum NSC content (expressed in glucose concentration) needed to generate the osmotic pressure (π) for the refilling of the gas-filled tracheids, in a wood sample of given size and given Ψ_xyl_ at recovery^54^. Given the PLC measured before re-irrigation (20%), the sapwood area occupied by tracheids (25%, calculated in our samples from cross sections), the wood density of samples (0.51 g cm^−3^) and the Ψ_xyl_ measured at recovery of hydraulics (−0.6 MPa, Fig. 4), and assuming that a π of −0.7 MPa (i.e. 0.1 MPa more negative than Ψ_xyl_; assumption value) was necessary to counterbalance xylem tensions and reclaim water, the corresponding minimum glucose content required to reverse embolism would be 5 mg g^−1^ of DW (see calculations in the supplementary material). Therefore, ca. 6 mg g^−1^ DW in total NSC content, that is the drop in NSC content observed in the wood of stressed plants from drought to recovery conditions in our study, should have been theoretically sufficient to provide the osmotic pressure required for the process.

In conifers, due to their wood anatomy, the occurrence and kinetics of refilling could depend on the amount and arrangement of ray parenchyma within the wood and on the distance between rays and embolized conduits^19^, because water must come from living cells. In the stems of spruce saplings used in our experiment we measured a xylem parenchyma fraction of about 6%, which is in line with the average value reported for conifers (8%^20^). In Norway spruce, parenchyma rays are well spread within the wood^20^ and, albeit present in low percentage, have been already proven to be capable of driving water into embolized tracheids upon needle water uptake, after winter embolism^25^. Therefore, it is likely that for this species wood parenchyma rays could also contribute to restore xylem functionality in summer when, after a drought spell, a rain event occurs and water in the soil becomes again available for the plant. In our experiment, the amount of embolized conduits was limited and probably low enough to allow for relatively rapid refilling. It is however possible that when a larger fraction of xylem area is embolized, refilling in conifers does not occur^33,34^ or is only partial or requires longer time and/or higher NSC availability.

## Conclusions

In our study we gave new insight on the capability of saplings of a conifer species, Norway spruce, to recover stem xylem hydraulic function after relief of soil drought. Hydraulic recovery was associated with NSC depletion, which was specifically localized in the stem sapwood fraction. Therefore, we discussed the possible causes of NSC depletion in the stem wood in the phase of recovery from drought: our hypotheses should be tested in further studies, especially on conifers and in field experiments. Separation of stem wood and bark fractions for NSC analysis has been a crucial expedient to reach our objectives and should be taken into consideration in future studies. The occurrence and kinetics of refilling at different thresholds of embolism should be studied, especially with the help of non-invasive imaging techniques. Given that aquaporins in VACs have been shown to promote refilling in conifer needles^26^, research on aquaporin expression in the parenchyma of conifers’ secondary xylem would be needed.

A high resilience to fluctuations in water availability may be a key-factor determining the success of some tree species in several forest ecosystems^38^. Applying multiple cycles of cavitation-refilling could also reveal whether progressive depletion of NSCs in the wood occurs and if it is connected to the refilling capability and its kinetics. In that case, the time needed to recover NSCs to control values after re-irrigation might be probably crucial. In the light of the predicted climate change scenarios, possible consequences of NSC consumption due to drought-recovery cycles on productivity, survival and general drought susceptibility should be taken into account.

## Methods

### Plant material and experimental design

The experiment was conducted at the Greenhouse Center Duernast (48°24′16.1″N; 11°41′34.5″E, Duernast, Germany). In April 2014, four years old Norway spruce (*Picea abies* (L.) Karst.) trees of South Bavarian origin (Hoermann Pflanzen GmbH, Schrobenhausen, Germany), were transplanted in 20 l cylindrical plastic pots. The substrate used for planting was a mixture of 70% forest loamy soil (upper 20 cm of a luvisol, collected from a local stand of spruce at Kranzberg Forest, Freising) and 30% sand. A slow-release fertiliser (Osmocote^®^, ICL Fertilizers Deutschland GmbH, Germany) was added to the soil and pesticides against aphids and fungi were sprayed on leaves at the beginning of the vegetation period. In spring 2015, a liquid fertilizer (Hakaphos^®^ Blau 15-10-15 + 2, COMPO Gmbh & Co., Germany) was added to the soil. The soil was maintained at field capacity by an automated drip irrigation system. Pots were placed in a greenhouse equipped with a retractable roof. During the vegetation period, the roof was left open during the day with exception of rainfall events, in order to assure natural direct sunlight to the plants and avoid overheating. Pots were periodically and randomly moved within the greenhouse space.

Air temperature (°C), relative humidity (RH, %) and Photosynthetic Photon Flux Density (PPFD, µmol m^−2^ s^−1^) were measured at tree height in the middle of the greenhouse and recorded every 10 min by a data logger (model DL2e, Delta-T Devices, Cambridge, UK).

The main results of the present study derive from measurements carried out through a drought-recovery treatment applied in summer 2015 (see below) on plants that were drought stressed the year before (summer 2014). This was done in order to test the long-term effects of the previous drought on hydraulics, water relations and stem NSC pool size. In both drought cycles, plants were subjected to water shortage until leaf minimum water potential values between −3.0 and −3.5 MPa were reached. Afterwards plants were re-irrigated at soil field capacity. These target water potentials corresponded to ca. 10–40% loss of hydraulic conductance, according to vulnerability curves of *P. abies* available in the literature^43,44^.

The first drought cycle started on 23 July 2014. 26 plants were kept watered at soil field capacity (C_1cycle_, control trees) and 25 underwent drought (D_1cycle_, drought trees), induced by withholding irrigation. After recovery, all plants were kept well-watered until the second drought treatment, which started the following summer (2015). During the first drought cycle (summer 2014), air temperature and relative air humidity averaged 18.8 °C and 80%, respectively. In winter 2014–2015, the minimum temperature reached in the greenhouse was −4.3 °C.

In the following summer, plants from C_1cycle_ and D_1cycle_ groups were randomly assigned to two groups that were, as for 2014, a control (kept well-watered at soil field capacity by drip-irrigation) and a drought treatment. Therefore, four groups were formed: CC (both years control; n = 10), CD (first year control, second year drought; n = 16), DC (first year drought, second year control; n = 9) and DD (both years drought; n = 16). During this second drought cycle period, maximum daily PPFD ranged between 340 and 1690 µmol m^−2^ s^−1^ relative humidity (RH) oscillated between 20 and 90% and mean daily air temperature was between 23 and 30 °C. Maximum daily temperatures ranged between 23 and 43 °C. In order to test possible long-term legacies of the 2014 drought, a first measurement campaign (“Pre-drought”) was carried out at the end of June 2015 (i.e. the week before the beginning of the second drought treatment), by measuring soil volumetric water content (SVWC), gas exchange, water potentials, stem PLC and NSC content. To this aim, four C_1cycle_ and four D_1cycle_ trees were harvested. Twigs and needles were fully expanded at the beginning of measurements. Drought started on 30 June and irrigation of CD and DD trees was regulated in order to reach the target minimum water potential almost at the same time in all trees, to avoid that different duration of the drought would affect NSC concentration. To this purpose, in the first four weeks of the treatment, trees were daily irrigated with half of their individual daily water consumption, which was measured before starting the experiment by weighing each drought pot over a 24 h time interval. Afterwards irrigation was completely withheld in order to reach the target leaf water potentials. Trees not harvested for PLC and NSC content at the end of drought (“End-drought” campaign) were re-irrigated to soil field capacity and then were kept well-watered by drip irrigation as control trees. After six/seven days of re-irrigation, trees were harvested for a third (“Recovery”) campaign. Plants to be harvested in each campaign were randomly selected already before the start of the experiment.

### Soil volumetric water content

Soil volumetric water content (SVWC, %) was measured weekly in all the pots, over the whole second drought cycle period, via time domain reflectometry (TDR 100, Campbell Scientific, Inc., Logan, Utah, USA). Probes 20 cm in length were inserted vertically into the pots for instantaneous measurements. A mean value over the total depth was given as output. Measurements started on 30 June 2015 (start of drought, all trees well-watered) and ended on 14 August 2015 (when all pots were re-irrigated after the drought treatment).

### Water potentials and gas exchange

Pre-dawn (Ψ_pd_) and midday leaf water potentials (Ψ_md_) were measured between 3:00 and 4:30 h and between 11:30 and 13:30 h (solar time), respectively, in current-year fully developed twigs with a Scholander-type pressure chamber (mod. 1505D, PMS Instrument co., Albany, USA). In the first drought cycle (2014), only Ψ_md_ were measured, on subsamples (n = 5) and on a weekly basis (see Supplementary Fig. S1). In both drought cycles, Ψ_md_ were measured more frequently when values were approaching the target for re-irrigation. In the three main campaigns of the second drought cycle (2015), xylem water potentials (Ψ_xyl_) were measured in parallel with Ψ_md_, on twigs which were wrapped in plastic cling and aluminium foil the evening before, in order to stop transpiration and allow for equilibration of water potentials^55^.

In 2015, in order to monitor the effects of the drought treatment on leaf gas exchange, CO_2_ assimilation rate (*A*, µmol m^−2^ s^−1^), stomatal conductance (*g*_s_, mmol m^−2^ s^−1^) and leaf transpiration (*E*, mmol m^−2^ s^−1^) were measured on 25 June (before starting the drought treatment), on 16 July and on 3 August (last week of drought). Measurements were carried out in sun-exposed twigs during sunny days (PPFD between 1100 and 1500 µmol m^−2^ s^−1^), between 11.30 and 13.30 h (solar time), with a portable gas analyser (Licor 6400, LI-COR Inc., USA). CO_2_ concentration was set to 400 ppm. Due to the limited size of spruce needles, a conifer chamber made of transparent plastic (6400-05 LI-COR Inc., USA; measurements under ambient light) was used on a current-year twig. In each campaign, measurements were carried out in three to eight trees per treatment, on one twig per tree. Gas exchange parameters were normalized by the total needle area of the twig segment included in the chamber. Due to the three-dimensional structure of spruce needles, total projected area was multiplied by a factor 3.2^56^.

### Stem radius variation

Stem radius variation was detected using diameter dendrometers (model DD-S, Ecomatik, Dachau/Munich, Germany), installed at the basal portion of the stem of two-three trees per treatment. From installation (March 2015) to tree harvest (August 2015), dendrometer signals were recorded every 10 min by the data logger used for environmental data (see above). The diameter variation, defined as the relative change in diameter with respect to the beginning of drought treatment (30 June), was calculated.

### Water potential isotherms

In order to assess possible drought-induced adjustments of turgor loss point, water potential isotherms^57^ (or pressure-volume curves) were measured before the beginning of the second drought cycle (end of June 2015) in C_1cycle_ and D_1cycle_ trees (n = 5) and after the second drought treatment (upon restored irrigation) in twigs of control and drought treatments (n = 5). Fully developed current-year twigs were detached in the early morning and allowed to rehydrate for 30 to 60 min (until Ψ > −0.2 MPa) while wrapped in plastic cling. After measuring initial water potential (Ψ) and mass, samples were progressively dehydrated on the bench and measurements were periodically repeated until a linear relation (R^2^ > 0.95) between water loss and Ψ^−1^ was obtained. Water potential at turgor loss point (Ψ_tlp_), osmotic potential at full turgor (π_0_) and bulk modulus of elasticity (ε) were determined using a spreadsheet tool^58^. From each twig used for pressure-volume curves, leaf mass per area (LMA, g m^−2^) was calculated dividing the total leaf dry mass by the total leaf projected area.

### Percentage loss of stem hydraulic conductance (PLC)

Percentage loss of stem conductance (PLC, %) was assessed in 2015 in the “Pre-drought” (29–30 June), “End drought” (8–12 August) and “Recovery” (16–20 August) campaigns. Trees were cut at the basal portion of the stem and then trimmed several times under water, removing lateral branches, until the selected portion (two-year old) of the main stem reached about 3–4 cm in length. Segments were debarked completely to avoid tracheid occlusion by resin. After final thin and sharp cuts at both ends, samples were connected to the Xylem Embolism Meter (XYL’EM - Plus, Bronkhorst, France). Hydraulic conductance measurements were performed at 25 °C under low water pressure (7 kPa) using degassed, filtered (0.2 µm) water with 10 mM KCl and 1 mM CaCl_2_ added^55,59^. Consecutive flushes of 10 min each were applied with the same solution at 0.1 MPa, until no further increase in conductance (maximum hydraulic conductance, k_max_) was detected. Percentage loss of hydraulic conductance (PLC) was calculated as:

$${\rm{PLC}}=100\,(1-{{\rm{k}}}_{{\rm{i}}}/{{\rm{k}}}_{{\rm{\max }}})$$

where k_i_ is the initial native hydraulic conductance (i.e. measured before flushes).

We are aware of the so called “Wheeler effect”, according to which cutting samples under tension, even if under water, produces artefactually higher PLC values^60^. However, we note that those results have been confuted by follow-up studies^61–65^ applying standard handling and sampling protocols. Moreover, we note that even Wheeler *et al*. (2013) could not find any cutting artefact in a short-vesselled species. In our case, trees were cut and trimmed several times under water, thus leading to relaxation of xylem tension. This, coupled to the fact that we are dealing with a tracheid-bearing species, make us reasonably confident about the lack of artefacts in our PLC measurements.

### Stem non-structural carbohydrate (NSC) analysis

A 4 cm long stem segment adjacent to the portion used for hydraulic conductance measurements was taken from each harvested tree (in each of the three campaigns). Bark (including cambium) was separated from wood with a razor blade and both portions were treated as separate samples, microwaving them three consecutive times at 700 W for 30 s to stop enzymatic activity. After oven-drying at 70 °C until constant mass, each sample was ball milled to fine powder, and 20 mg of dry mass was used for soluble sugars extraction in distilled water. A first extraction was carried out in 1 ml water and other two subsequent ones in 0.5 ml water^66^, incubating the suspension for 10 min in 80 °C water bath. The remaining dry pellet from soluble sugars extraction was re-suspended in 1.0 ml distilled water and starch was hydrolysed to glucose using heat-stable α-Amylase from *Bacillus licheniformis* (1250 U/ml, 30 min incubation at 80 °C) and amyloglucosidase from *Aspergillus niger* (3 U/ml, overnight incubation at 37 °C). After centrifugation, final extracts were filtered (0.45 µm nylon filters) and stored at −20 °C until analysis. NSC analysis was performed with high-pressure liquid chromatography (Schambeck SFD, Bad Honnef, Germany) equipped with a Carbosep CHO-820 Ca^2+^ column (Transgenomic, Glasgow, UK), maintained at 90 °C. Millipore water was used as mobile phase at a flow rate of 0.5 ml min^−1^ ^67^. Sugar specimens (glucose, fructose, sucrose, pinitol, galactose, stachyose and raffinose) were identified by retention time and concentration was quantified by comparing peak heights in chromatograms with calibration curves obtained from standard solutions. Starch content was quantified as glucose equivalents. Total NSC content was considered the sum of all measured specimens: starch, glucose, fructose and sucrose constitute the major physiologically important carbon storage compounds; the others are mainly synthetized in response to stresses like drought^48^.

### Aboveground biomass

In the “End-drought” and “Recovery” campaigns (August 2015) the aboveground biomass of each tree harvested for PLC measurements was measured after being oven dried at 70 °C for 48 h. For data analysis, the two campaigns were considered as a single one.

### Statistical analysis

Analyses were carried out at a probability level of *P* < 0.05, using R (v. 3.1.2, R development Core Team, 2014). All data were tested for normality (Shapiro-Wilk test) and homoscedasticity (Bartlett test) and, whenever necessary, log-, square root- or exponential- transformed.

Two-sided welch t-test was used to test differences between C_1cycle_ and D_1cycle_ trees in all parameters measured before starting drought (“Pre-drought” campaign), except for PLC that was tested with Mann-Whitney U-test (non-normality).

Differences between treatments for *g*_s_, *E*, Ψ_pd_, Ψ_xyl_ and Ψ_md_ were tested per campaign with ANOVA followed by post-hoc Tukey-HSD. Differences in *A* and PLC (non-normality) were tested in each campaign with the non-parametric Kruskal-Wallis test followed by Conover’s-post-hoc (R package pmCMR).

In the “End-drought” and “Recovery” campaigns, the influence of the first drought cycle, second drought cycle and of their interaction on stem NSC content was tested using two-way ANOVA. As the first drought cycle did not significantly affect NSCs (Table S3), data of the “End-drought” and “Recovery” campaigns were pooled per water regime (well-watered treatments: CC & DC and drought treatments: CD & DD) and a two-way ANOVA (treatment and campaign as factors) followed by Tukey HSD post-hoc test were performed. A two-way factorial ANOVA (first year and second year treatments as factors) followed by Tukey HSD test was used to test differences in aboveground biomass between treatments.

Correlations PLC-starch and PLC-total soluble at the end of drought were tested calculating Spearman’s rank correlation coefficients.

### Data availability

The datasets generated during and/or analysed during the current study are available from the corresponding author on reasonable request.

## Electronic supplementary material

Supplementary information
