# Supplementary material for: Post-drought hydraulic recovery is accompanied by non-structural carbohydrate depletion in the stem wood of Norway spruce saplings
Source: Sci Rep. 2017 Oct 30;7:14308. doi: 10.1038/s41598-017-14645-w (PMC5662761; doi:10.1038/s41598-017-14645-w)
Supplement: Supplementary file 1 — Supplementary information [file 41598_2017_14645_MOESM1_ESM.pdf]

Manuscript title

Post-drought hydraulic recovery is accompanied by non-structural carbohydrate depletion in the stem wood of Norway spruce saplings

Author list

Martina Tomasella, Karl-Heinz Häberle, Andrea Nardini, Benjamin Hesse, Anna Machlet, Rainer Matyssek

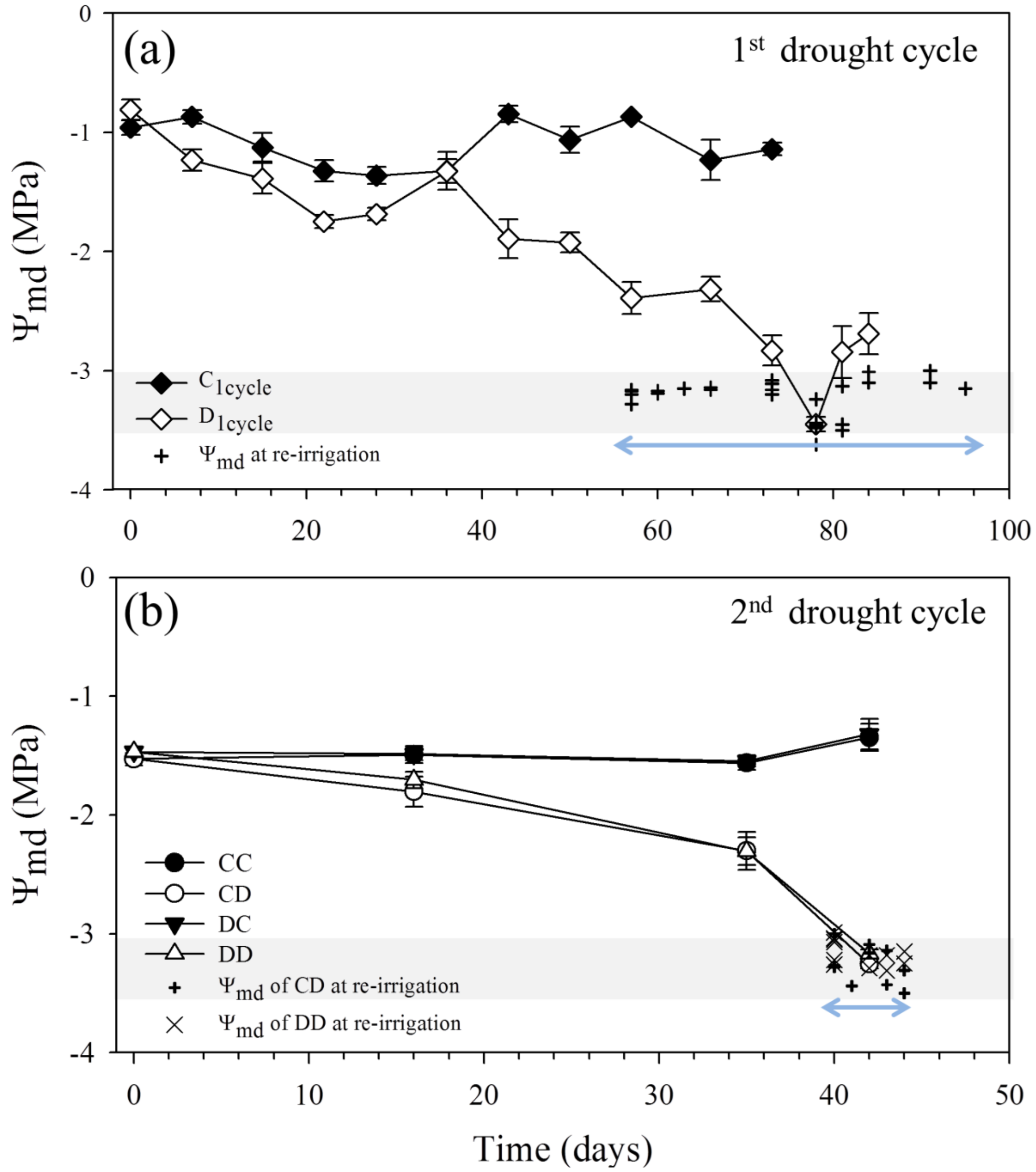

Figure S1. **Changes in midday water potentials (Ψ_{md}) over the first and second drought cycles**. **(a)** Ψ_{md} monitored over the first drought cycle (summer 2014) in well-irrigated (control, C_{1cycle}, closed symbols) and drought stressed (D_{1cycle}, open symbols) trees, from the time when irrigation was withheld (Day 0). **(b)** Ψ_{md} monitored over the second drought cycle (summer 2015) in CC (control in 2014 and 2015), CD (control in 2014, drought in 2015), DC (drought in 2014, control in 2015) and DD (drought in 2014 and 2015) trees, from the time when irrigation was reduced (Day 0). Note the different time scales in (a) and (b). Symbols denote means and bars are standard errors, while crosses indicate for each D_{1cycle} individual the Ψ_{md} at re-irrigation. The shaded horizontal area highlights the target range of Ψ_{md} for re-irrigation of drought-stressed trees and the blue arrow indicates the time period when plants were re-irrigated.

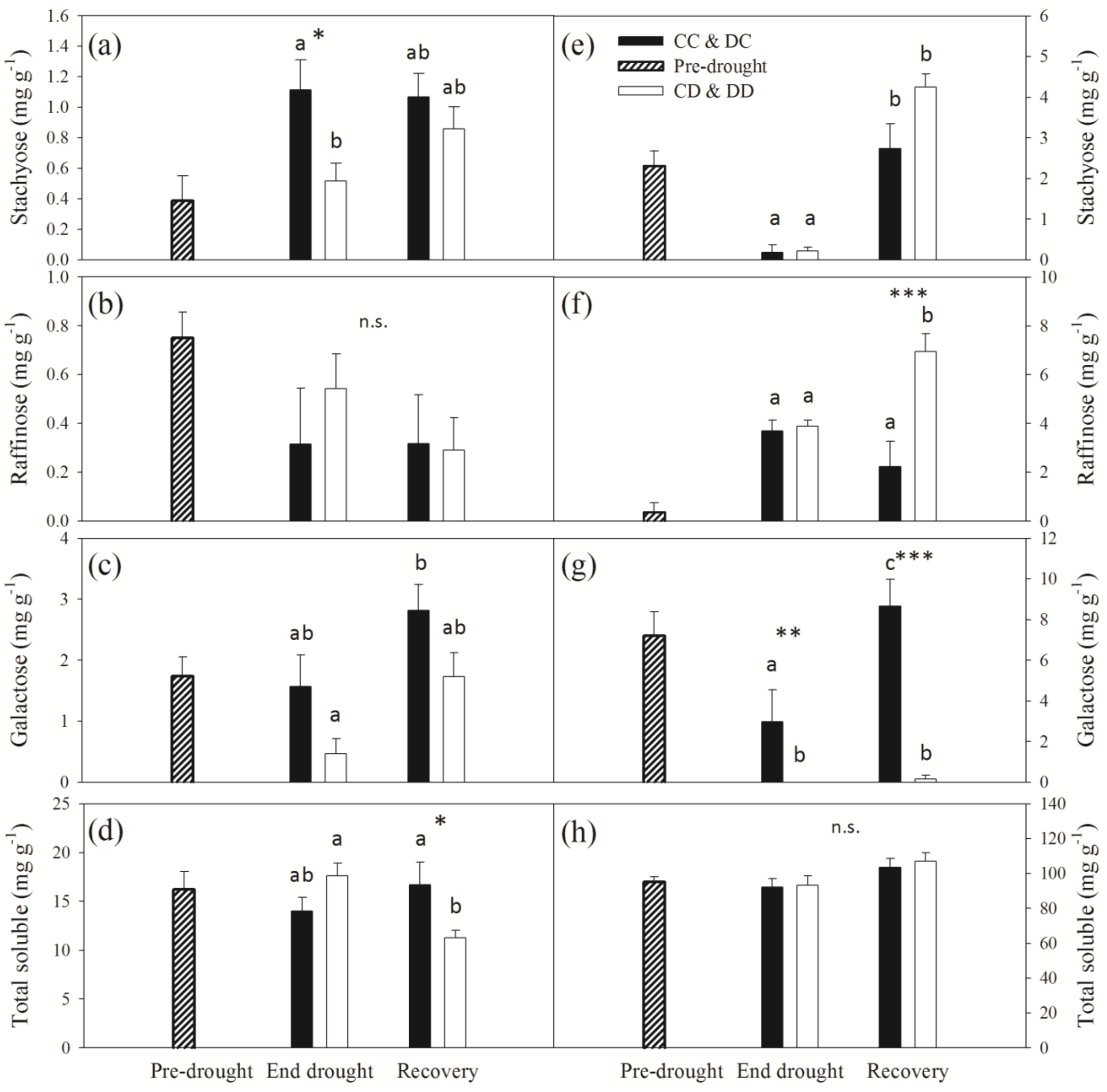

Figure S2. Additional non-structural carbohydrates (NSC) measured in stems during the second drought cycle (2015). (a-c) NSC concentration (in mg g^{-1} of dry mass) in stem wood and **(d-f)** bark, measured in "Pre-drought", "End drought" and "Recovery" campaigns. "Total soluble" is the sum of all soluble sugars measured (sucrose, fructose, glucose, pinitol, stachyose, raffinose and galactose). In the "Pre-drought" campaign, data were pooled in one single group. In the "End drought" and "Recovery" campaigns, well-watered (CC and DC) as well as drought (CD and DD) treatments were pooled, resulting in one control (CC & DC) and one drought (CD & DD) group. Please note the different scales between wood and bark NSCs content. Bars are means ± standard error (n = 6-12). Different letters indicate significant differences between treatments and campaigns (two-way ANOVA and Tukey-HSD, only data of "End drought" and "Recovery" are compared). n.s. = no significant difference. Asterisks denote the significance of differences among treatments within a given campaign (*$0.01<P<0.05$, **$0.001<P<0.01$, ***$P<0.001$).

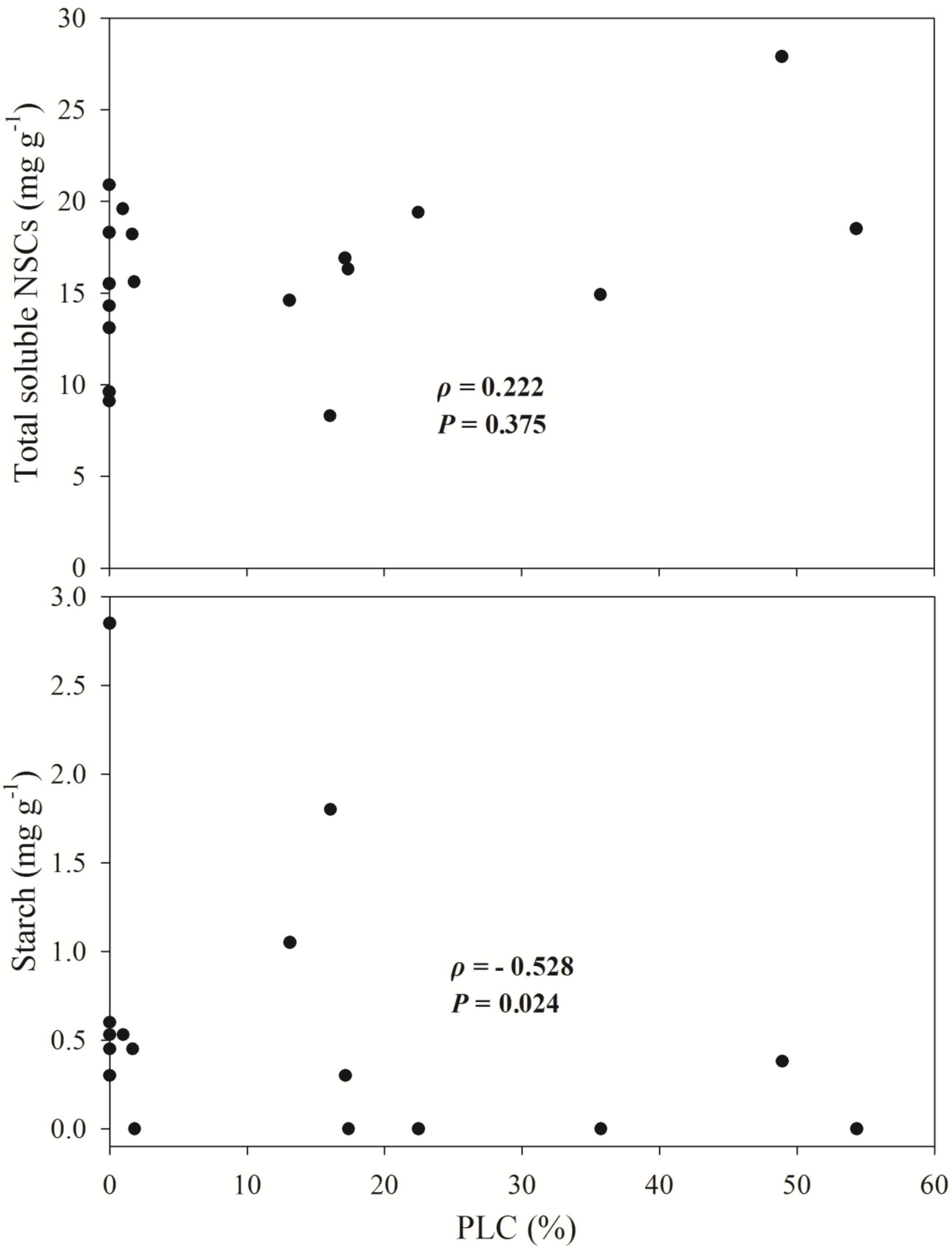

Figure S3. The relationship between stem wood non-structural carbohydrates (NSCs) and PLC at the end of the second drought cycle ("end drought" campaign). The correlation between percentage loss of xylem hydraulic conductance (PLC) and total soluble NSCs **(a)** or starch content **(b)** measured in the stem wood in the "end-drought" campaign (summer 2015). The Spearman correlation coefficient (ρ) and *P*-value (*P*) are reported.

Table S1. Parameters measured under well-watered conditions before the beginning of the second drought cycle (summer 2015). Pre-dawn (Ψ_{pd}), xylem (Ψ_{xyl}) and midday (Ψ_{md}) water potential, CO_2 assimilation rate (*A*), stomatal conductance (g_s), leaf transpiration (*E*), water potential at turgor loss point (Ψ_{tlp}), osmotic potential at full turgor (π_0), bulk modulus of elasticity (ε), leaf mass per area (LMA), percentage loss of xylem hydraulic conductance (PLC) and stem non-structural carbohydrates measured at the end of June 2015 in control (C_{1cycle}) and drought (D_{1cycle}) treatments from the first drought cycle. NSC specimens were measured separately in wood and bark. "Total soluble" is the sum of all soluble NSCs. Values are means ± standard error. Number of replicates (*n*) and *P*-value from Welch's t-tests are given.

Parameter		C_{1cycle}	D_{1cycle}	*n*	*P*-value
Ψ_{pd} (MPa)		-0.41 ± 0.08	-0.40 ± 0.06	7	0.99
Ψ_{xyl} (MPa)		-1.11 ± 0.07	-0.86 ± 0.04	7	0.01
Ψ_{md} (MPa)		-1.53 ±0.05	-1.47 ± 0.04	7	0.40
A (µmol m^{-2} s^{-1})		1.71 ± 0.32	2.18 ± 0.34	7	0.34
g_s (mmol m^{-2} s^{-1})		14.5 ± 2.9	19.5 ± 3.0	7	0.26
E (mmol m^{-2} s^{-1})		0.82 ± 0.15	0.96 ± 0.12	7	0.46
Ψ_{tlp} (MPa)		-1.57 ± 0.10	-1.58 ± 0.15	5	0.94
π_0 (MPa)		-1.39 ± 0.11	-1.42 ± 0.14	5	0.90
ε (MPa)		26.8 ± 5.9	32.4 ± 9.6	5	0.63
LMA (g m^{-2})		146 ± 10	165 ± 15	5	0.32
PLC (%)		2.6 ± 2.6	0.62 ± 0.62	4	1.00
Starch (mg g^{-1})	wood	2.14 ± 0.42	1.29 ± 0.43	4	0.21
	bark	17.85 ± 2.93	18.37 ± 7.13	4	0.95
Sucrose (mg g^{-1})	wood	3.50 ± 1.05	4.20 ± 0.51	4	0.58
	bark	35.75 ± 2.21	36.8 ± 2.21	4	0.75
Fructose (mg g^{-1})	wood	3.35 ± 0.58	2.50 ± 0.47	4	0.30
	bark	14.07 ± 1.51	16.20 ± 2.02	4	0.43
Glucose (mg g^{-1})	wood	2.75 ± 0.57	2.00 ± 0.13	4	0.28
	bark	11.77 ± 2.11	12.82 ± 2.33	4	0.75
Pinitol (mg g^{-1})	wood	3.82 ± 0.99	4.55 ± 0.86	4	0.60
	bark	20.62 ± 1.89	22.40 ± 2.20	4	0.56
Stachyose (mg g^{-1})	wood	0.20 ± 0.20	0.57 ± 0.25	4	0.28
	bark	1.87 ± 0.64	2.75 ± 0.27	4	0.28
Raffinose (mg g^{-1})	wood	0.75 ± 0.13	0.75 ± 0.18	4	1.00
	bark	0.00 ± 0.00	0.75 ± 0.75	4	0.39
Galactose (mg g^{-1})	wood	1.95 ± 0.25	1.52 ± 0.62	4	0.56
	bark	8.82 ± 0.82	5.57 ± 2.01	4	0.21
Total soluble (mg g^{-1})	wood	16.32 ± 3.32	16.10 ± 2.16	4	0.96
	bark	92.92 ± 2.85	97.30 ± 10.89	4	0.80
Total NSCs (mg g^{-1})	wood	18.46 ± 3.73	17.39 ± 1.75	4	0.81
	bark	110.77 ± 4.32	115.67 ± 11.95	4	0.72

Table S2. Water potential isotherm parameters measured at re-irrigation in the second drought cycle (summer 2015). Water potential at turgor loss point (Ψ_{tlp}), osmotic potential at full turgor (π_0), bulk modulus of elasticity (ε) and leaf mass per area (LMA) measured the week after re-irrigation, in the second drought cycle, in twigs of control (i.e. well-irrigated, CC and DC) and drought (CD and DD) plants. Values are means ± standard errors and *P*-values from Welch's t-test are given.

	CC & DC	CD & DD	*P*-value
Ψ_{tlp} (MPa)	-1.79 ± 0.03	-1.99 ± 0.11	0.16
π_0 (MPa)	-1.56 ± 0.01	-1.72 ± 0.11	0.21
ε (MPa)	30.0 ± 18.8	21.2 ± 3.2	0.82
LMA (g m^{-2})	154 ± 14	169 ± 6	0.42

Table S3. Effect of the two drought cycles on non-structural carbohydrate (NSC) content. *P*-values from two-way ANOVA examining the effect of the first drought cycle (first), the second drought cycle (second) and their interaction (first*second) on non-structural carbohydrate (NSC) content (measured separately in stem wood and bark) in the "end-drought" and "recovery" campaigns performed in the second drought cycle. "Total soluble" is the sum of all soluble NSCs. *P* <0.05 indicate significant effects and are formatted in bold.

		End-drought (2015)			Recovery (2015)		
		first	second	first*second	first	second	first*second
Starch	wood	0.13	0.12	0.94	0.58	0.11	0.94
	bark	0.60	**<0.001**	0.28	0.51	0.67	0.10
Sucrose	wood	0.73	0.28	0.30	0.83	**0.02**	0.98
	bark	**0.03**	0.16	0.58	0.48	0.69	0.29
Fructose	wood	0.67	0.56	0.20	0.54	0.33	0.40
	bark	0.44	**0.04**	0.40	0.24	0.06	0.87
Glucose	wood	0.90	0.89	0.37	0.50	**0.01**	0.31
	bark	0.53	0.24	0.47	0.74	0.98	0.63
Pinitol	wood	0.67	**<0.001**	0.58	0.05	0.10	0.77
	bark	0.38	0.88	0.15	0.24	0.09	0.99
Stachyose	wood	0.61	**0.02**	0.18	0.76	0.34	0.52
	bark	0.35	0.76	**0.01**	0.35	**0.04**	0.76
Raffinose	wood	0.96	0.27	0.50	0.49	0.90	0.10
	bark	0.11	0.87	0.32	0.78	**0.003**	0.28
Galactose	wood	0.42	0.07	0.96	0.07	0.08	0.85
	bark	0.74	**0.03**	0.67	0.97	**<0.001**	0.18
Total soluble	wood	0.75	0.15	0.69	0.10	**0.01**	0.47
	bark	0.10	0.95	0.57	0.69	0.66	0.31
Total NSCs	wood	0.99	0.16	0.73	0.07	**0.005**	0.39
	bark	0.28	0.26	0.35	0.80	0.64	0.51

Calculation of theoretical minimum glucose concentration necessary to generate enough osmotic pressure for refilling of embolized tracheids in the measured spruce samples

Percentage loss of xylem conductivity (PLC) before re-irrigation = 20%

Percentage loss of xylem conductivity (PLC) after recovery = 0%

Ψ_{xylem} after refilling = -0.62 MPa -> Required osmotic pressure ~ -0.7 MPa

Assumed a sample of diameter = 0.8 cm and length (L)= 4 cm (volume = 2 cm^{-3}).

Area cross section (A_{tot}) = πr^2 = 50.2 mm^2

Area pith (A_{pith}) = πr^2= 1.1 mm^2

Sapwood area (A_{wood}) = A_{tot} - A_{pith}= 49.1 mm^2

Volume wood = A_{wood} x L = 49.1 x 40 = 1964 mm^3 = 1.964 cm^3= 1.964 ml

The sapwood area occupied by tracheids in spruce samples (from anatomical analysis) is about 25% and PLC recovered was 20%, therefore the volume of gas filled tracheids (V_{trach}) to be refilled was:

V_{trach} = 1.964 x 0.25 x 0.20= 0.0982 ml

Assuming a temperature (T) of 293 K, on the basis of the Van't Hoff's equation:

Solute concentration (C_s) = $\pi/(R*T)$ = 0.7 /(8.314x10^{-3} x 293)= 0.287 mol l^{-1}

Therefore in 0.0982 ml there are 2.82 x 10^{-5} mol of solutes. Molar mass of glucose = 180 g mol^{-1}

2.82 x 10^{-5} * 180 = 5.08 mg= mass of glucose in the sample

The wood density of spruce samples was 0.51 g cm^{-3}, therefore the dry mass of a 2 cm^{-3} sample is 1.02 g.

Glucose concentration required for refilling= 5.08/1.02= 5.0 mg g^{-1}
